# Cognition, Statins, and Cholesterol in Elderly Ischemic Stroke Patients: A Neurologist’s Perspective

**DOI:** 10.3390/medicina57060616

**Published:** 2021-06-13

**Authors:** Anamaria Jurcau, Aurel Simion

**Affiliations:** 1 Department of Psycho-Neurosciences and Rehabilitation, Faculty of Medicine and Pharmacy, University of Oradea, nr 1 Universitatii Street, 410087 Oradea, Romania; aurel.simion1962@gmail.com; 2 Neurology Ward, Clinical Municipal Hospital “dr. G. Curteanu”, nr 12 Corneliu Coposu Street, 410469 Oradea, Romania; 3 Neurological Rehabilitation Ward, Clinical Municipal Hospital “dr. G. Curteanu”, nr 12 Corneliu Coposu Street, 410469 Oradea, Romania

**Keywords:** cholesterol, LDL, cognitive impairment, ischemic stroke

## Abstract

*Background and Objectives:* The efficacy of hydroxy methyl glutaryl-coenzyme A reductase inhibitors (statins) in reducing the incidence of cardiovascular events pushed the target LDL-cholesterol (LDL-C) levels lower and lower in successive guidelines despite signals regarding potential cognitive side effects. We evaluated the relationship between cognitive impairment and LDL-C levels in elderly ischemic stroke patients. *Materials and Methods:* 29 ischemic stroke patients aged 65 and above with LDL-C levels ≤70 mg/dL, classified according to the TOAST criteria, underwent detailed neuropsychological testing comprising the MMSE test, Montreal Cognitive Assessment (MoCA) and Addenbrooke’s Cognitive Evaluation (ACE-III) test. Their performances were compared to those of 29 age-matched ischemic stroke patients with LDL-Cl levels >71 mg/dL. *Results:* The MMSE test failed to detect significant cognitive differences between the two groups. The MoCA and ACE-III tests detected impairments in visuo-spatial/executive function, attention, and recall/memory in patients with low LDL-C. A stepwise linear regression model of the ACE-III total scores revealed that LDL-cholesterol levels could contribute to 13.8% of the detected cognitive dysfunction, second in importance only to age, which contributed to 38.8% of the detected impairment. *Conclusions:* Physicians should be cautious when prescribing statins to elderly people. Hydrophilic ones may be preferred in cognitively impaired patients.

## 1. Introduction

The involvement of cholesterol in cardiovascular disease pathogenesis has been known since the second half of the 20th century, but the dietary measures and poorly tolerated available medications used to reduce cholesterol levels, such as cholestyramine and clofibrate, did not gain popularity among physicians [1].

The release of the results of the Scandinavian Simvastatin Survival Study (4S) in 1994 [2] followed by other studies reinforcing the beneficial effects of statins both in primary and secondary prevention of cardiovascular disease [3,4,5] changed these practices. In the past decades we witness a dramatic change in the management of dyslipidemia [6]. 

The first Adult Treatment Panel for National Cholesterol Education Program (NCEP-ATP I) recommended in 1988 low-density cholesterol (LDL-C) target level of <130 mg/dL for primary prevention of cardiovascular disease [7]. Subsequent guidelines gradually lowered the target LDL-C levels and further defined the risk groups of vascular events until in 2019 the European Society of Cardiology recommended an LDL-C target of ≤55 mg/dL and a ≥50% LDL-C reduction from baseline in very-high-risk patients [8], a recommendation ranked as class I level of evidence A for secondary prevention and as class I level of evidence B in primary prevention, mentioning that older people should be treated in the same way as younger patients (class I, level of evidence A). For stroke as well, the European Stroke Organization (ESO) endorsed aggressive lipid-lowering therapy [9] recommending standard treatment with atorvastatin 80 mg/day in secondary prevention after an ischemic stroke or a transient ischemic attack (Grade A). 

In North America, the 2006 the American Stroke Association guideline recommended the management of dyslipidemia in stroke patients according to primary prevention guidelines [10] but after the results of the SPARCL trial were released [11] the American Heart Association 2008 guidelines recommended intensive statin therapy in patients with ischemic stroke setting as treatment goal LDL-C levels below 100 mg/dL [6]. The 2018 guidelines pushed further, recommending a ≥50% reduction in LDL-C levels (class I, level of evidence A) or absolute levels of LDL-C below 70 mg/dL [12], a target reinforced by the Treat Stroke to Target trial which reported fewer major cardiovascular events in patients with LDL-C levels below 70 mg/dL as compared to patients with LDL-C between 90 and 110 mg/dL [13].

Adding newer agents, such as ezetimibe [14] or monoclonal antibodies which inhibit proprotein convertase subtilisin/kexin type 9 (PCSK-9) inhibitors such as evolocumab or alirocumab allowed for more aggressive reduction of the LDL-C levels with further reduction of the cardiovascular risks [6]. The FOURIER trial showed that adding evolocumab reduced the LDL-C levels to a median of 30 mg/dL and thereby reduced the risk of stroke by 78% (number needed to treat 250) and the risk of stroke or transient ischemic attack by 80% (number needed to treat 200) during a mean follow-up period of 2.2 years [15]. These new agents have already entered guidelines, leading the ESO to state that PCSK9 inhibitors may be added to statins plus/minus ezetimibe to achieve the targeted very low LDL-C levels (Grade B). In addition, the ESO recommends adding evolocumab to statins in patients with stable cardiovascular disease and the combination of alirocumab with statin therapy in patients with acute coronary syndrome to reduce the risk of ischemic stroke (Grade A). New emerging lipid-lowering therapies to be included in guidelines in the future are inclisiran, a long active RNA interface that inhibits PCSK-9, a cholesteryl ester transfer protein inhibitor anacetrapib, or angiopoietin-like gene inhibitors [6,16,17,18].

Although reported side effects of statins are mainly myalgias, myopathies, elevations in liver enzymes, as well as a modest increase in the incidence of diabetes mellitus [19,20], the clinical trials on which the aforementioned recommendations were based were not designed to detect cognitive impairment. Post-marketing reports drew attention upon a transient cognitive impairment caused by statins in some patients, which prompted the Food and Drug Administration (FDA) to issue in 2012 a warning regarding the potential for reversible cognitive impairment in statin users [21]. 

The relationship between statin use and the risk of developing dementia (Alzheimer’s disease or all-cause dementia) is, however, more complex. A number of cohort studies or meta-analyses concluded that long-term statin use can reduce this risk or reduce dementia progression [22,23,24,25]. However, many of these studies did not analyze the achieved total cholesterol and LDL-C levels [22], found significant reductions of the risk for Alzheimer’s disease but not for vascular dementia [23], or showed risk reduction for dementia with low doses of statins, but not for medium or high doses [26]. Similarly, Poly et al. concluded that statin therapy should remain restricted for cardio-vascular disease [23]. 

Obviously, these findings raise the question of “how low can we go” with LDL-C reduction without significant side effects? The aim of the present study was to evaluate possible associations between HMG CoA reductase inhibitor therapy, levels of LDL-C and cognitive impairment in elderly patients.

## 2. Materials and Methods

### 2.1. Patients

The patients were selected from the ischemic stroke patients hospitalized at the Neurology ward of the Clinical Municipal Hospital “dr. G Curteanu” Oradea between 1 November 2017 and 31 January 2019. Inclusion criteria were:

- Age between 65 and 80 years;
- LDL-C levels below 70 mg/dL (this level was selected to comply with the currently available guidelines for the treatment of stroke);
- Hospitalization for acute ischemic stroke.

Exclusion criteria were:

- The presence of speech difficulties or aphasia, or of significant motor deficits which could interfere with a detailed neuropsychological testing;
- Prior diagnosis of dementia;
- Associated comorbidities which could interfere with cognitive function, like psychiatric conditions, hypothyroidism, Parkinson’s disease.

A number of 35 patients meeting the inclusion criteria were identified, but only 32 gave informed consent and 29 patients completed the evaluations (3 patients withdrew).

A group of 32 age-matched ischemic stroke patients with LDL-cholesterol levels above 70 mg/dL hospitalized during the same time period and having strokes of comparable severity consented to complete the same neuropsychological evaluations. The pairs of the patients lost to follow-up were excluded, resulting in a control group of 29 patients. 

### 2.2. Methods

Demographic factors like age, gender, as well as data on the medications used, particularly of statins, were recorded. Hypertension was diagnosed according to the JNC VIII criteria [27] and was considered present if the patient met these criteria or was on current antihypertensive treatment. The systolic and diastolic blood pressures recorded were the mean values of these measured each morning during hospitalization. 

Diabetes mellitus was diagnosed according to the current diagnostic criteria [28] or recorded if the patient was on current antidiabetic treatment.

Fasting blood samples were drawn the morning after hospital admission for measurement of the LDL-cholesterol, high-density lipoprotein cholesterol (HDL-C) HDL-cholesterol and triglyceride levels. 

Ischemic stroke subtype was diagnosed according to the TOAST criteria [29] and the neurological deficit scored on the National Institute of Health Stroke Scale (NIHSS) on admission. All patients were treated and evaluated according to the ischemic stroke protocol on evaluation and treatment applicable in our hospital. Aside from these evaluations, the patients included in both groups underwent a neuropsychological evaluation described below. 

Neuropsychological evaluation was carried out starting with day 7+/−2 from stroke onset (during hospitalization for acute ischemic stroke) by a psychologist certified in clinical psychology, who had no access to the patients’ chart and was not informed on the patients’ cholesterol levels. Three screening tests were administered:

- The MMSE test, a 30-point questionnaire which has been widely used in clinical as well as in research setting to screen for dementia and evaluate cognitive impairment [30].
- The Montreal Cognitive Assessment (MoCA) version 7.1, a one-page 30-point screening test used for detecting cognitive impairment [31]. MoCA scores range between 0 and 30. A correction of 1 point for education level below 12 years was applied according to the recommendations. A score of 26 or above was considered to be normal.
- The Addenbrooke’s Cognitive Examination—ACE-III -test, which tests five cognitive domains: attention/orientation, visuospatial skills, memory, language, and verbal fluency. It is scored out of 100, a higher score indicating better cognitive function. The recommended cut-off score is 88 [32].

Since these tests contain overlapping items, they were administered on separate days. 

### 2.3. Statistical Analysis

Statistical analyses were performed with the IBM SPSS v 24.0 software (IBM Corp., Armonk, NY, USA). Means of continuous variables were compared with the independent samples *t*-test and frequencies of categorical variables with the chi-square test. Correlations between different variables were performed both as bivariate ones and as partial correlations controlling for age and stroke severity as assessed by the NIHSS score. Finally, a stepwise hierarchical regression was conducted with age, body mass index, diastolic blood pressure, LDL-C, high density lipoprotein cholesterol (HDL-C) levels, and NIHSS score as independent variables and cognitive performance (Addenbrooke’s Cognitive Examination test total score) as dependent variable. Statistical significance was set at *p* < 0.05. 

## 3. Results

### 3.1. Demographic and Clinical Characteristics of the Two Groups of Patients

The age of the patients ranged from 65 to 82 years, with a mean of 73.24 +/− 5.22 years in both groups. 

Overall, there were more male patients than women included in the study, with 16 male patients (55.2%) in the low LDL-C group and 17 male patients (58.6%) in the high LDL-C group, without statistically significant differences in the gender distribution of the two groups. 

There were significantly more hypertensive patients in the control group (21 hypertensives and 8 normotensives) as compared to the patients with low LDL-C levels /dl (17 hypertensive patients and 12 normotensive ones, *p* = 0.014). The mean systolic blood pressure (BP) in the low LDL-C group was 150.62 +/− 22 mm Hg, while in the control group it was 160.76 +/− 22.9 mm Hg, with no statistical significance shown by the independent samples t-test. There were, however, significant differences in the diastolic BP, which was 76.14 +/− 7.65 mm Hg in the study group and 83.31 +/− 10.4 mm Hg in the control group (*p* < 0.05). 

Patients in both groups had similar prevalence of diabetes mellitus (Table 1), while patients with low LDL-C had significantly lower mean body mass indexes as compared to the control group: 23.9 +/− 5.53 vs. 26.5 +/− 4.35 kg/m^2^, *p* < 0.05. There were significant differences in the nutritional status of the patients included in the two groups, with 34.5% of patients being of low weight, 17.2% with normal weight, 31% overweight and 17.2% obese in the low LDL-C group and 3.4% of patients of low weight, 44.8% of normal weight, 24.1% overweight, and 27.6% obese in the high LDL-C group. 

A summary of these characteristics is provided in Table 1. 

### 3.2. Ischemic Stroke Subtypes

Although the small number of cases was not enough for the difference to reach statistical significance, patients with low LDL-C levels presented more embolic strokes as compared to the patients with higher LDL-C levels, who were more prone to developing thrombotic strokes while lacunar strokes and ischemic strokes of undetermined etiology had largely similar incidence, as shown in Table 2.

### 3.3. Lipid Profile

Due to the selection of the patients, the lipid profile showed significant differences between the two groups of patients, as summarized in Table 3. 

As expected, 86.2% of the patients in the low LDL-C group used statins, 55% of them atorvastatin, as opposed to the control group in which only 62.1% of patients were on prior statin therapy. 

### 3.4. Neuropsychological Evaluation

#### 3.4.1. MMSE Scores

The two groups of patients achieved similar MMSE scores, with a mean of 26 in the low LDL-C group and 26.86 in the high LDL-C group. 

#### 3.4.2. Montreal Cognitive Assessment (MoCA) Scores and Subscale Scores

Significant differences between the two groups of patients were found in the MoCA total score as well as in the visuo-spatial/executive, attention, and recall subscale scores, as shown in Table 4. 

There were no significant gender differences, either among the cognitively impaired patients or among those with normal cognition. The only subscale on which women performed significantly worse was the abstraction subscale (mean score 1.28 in females vs. 1.70 in males, *p* = 0.003).

#### 3.4.3. Addenbrooke’s Cognitive Examination—ACE-III Test Scores and Subscale Scores

The subscale scores and total scores obtained by the two patient groups are shown in Table 5. Patients with low LDL-C levels performed significantly worse on all subscales. Using 88 as cut-off point, the ACE-III revealed that 84.5% of patients showed cognitive disturbances. No significant gender differences were observed in the ACE-III total scores or subscale scores. 

### 3.5. Correlations

In a bivariate correlation analysis, the MMSE score, MoCA total score, and ACE-III total score showed to be significantly correlated with the age of the patients (*p* < 0.001), nutritional status (*p* < 0.001), LDL-C levels (*p* < 0.002), diastolic and systolic blood pressure. Neither gender, nor the presence of diabetes mellitus, triglyceride levels, or HDL-cholesterol levels were significantly correlated with these cognitive scores. Further, in a partial correlation analysis controlling for age and NIHSS score, the cognitive performance appeared to be significantly correlated with the LDL-C levels, and BMI, as shown in Table 6.

Finally, in a stepwise linear regression model using the adjusted total Addenbrooke’s scale total score as dependent variable and age, BMI, diastolic blood pressure, LDL-C, and NIHSS score as independent variables (Table 7), it appeared that age contributed to about 38% decrease in the total ACE-III score (R square change 0.388, *p* < 0.001), followed by a further 9% impairment (R square change 0.09) caused by poor nutritional status (low BMI), while low LDL-C levels could worsen the cognitive status by 13.8% (R square change 0.138, *p* < 0.001). Neither neurological deficit (NIHSS score), nor blood pressure or HDL-C levels were significantly related to cognitive impairment (*p* > 0.05)

## 4. Discussions

Our findings suggest that it is not unusual to find elderly patients with low.

LDL-C levels. This might be due to prolonged statin use caused by prior vascular events or social and economic particularities (low incomes, patients living alone) [33,34]. 

In our small group, patients with LDL-C levels below 70 mg/dL were more likely to develop cardioembolic strokes caused by atrial fibrillation (AF). Although AF and atherosclerosis share common risk factors such as hypertension, left ventricular hypertrophy, or obesity [6], the relationship between LDL-C and AF is rather inverse, in that high LDL-C levels seem to be associated with lower incidence of AF, as suggested by the analysis of participants in the ARIC study [35]. Even if functional outcome and survival is improved by statins in patients with cardioembolic strokes [36,37], this effect may be related to the neuroprotective and anti-inflammatory effects [38,39] of statins as well as to the augmentation of collateral blood flow [40,41], since evidence of any preventive effect of stroke recurrence is scarce [6]. 

Cholesterol is an important constituent of the brain, occurring in an unesterified form [42] as component of the myelin sheaths synthesized by oligodendrocytes (70%) and in the constitution of the plasma membranes of brain cells (30%) [43]. Because cholesterol entering the central nervous system is prevented by the blood brain barrier, the whole amount of cerebral cholesterol is synthesized by the brain through the mevalonate pathway [42]. Cholesterol synthesis, although significantly decreased after completion of myelination, continues in the adult brain at a basal level occurring mainly in the astrocytes, although neuronal synthesis and reutilizing cholesterol resulting from neuronal death have also been described [44]. Among the crucial roles of cholesterol in the CNS are modulating membrane fluidity and permeability [45] and regulating the activity of ion channels and neurotransmitter receptors, which are proteins residing in cell membranes [46]. Research has demonstrated that cholesterol is concentrated in microdomains, called lipid rafts [42] and is particularly abundant at synapses [47]. Depletion of cholesterol at pre- and postsynaptic membranes impairs exocytosis of synaptic vesicles [48] and alters the affinity of receptors to neuromediators, which could lead to impaired synaptic plasticity and interfere with learning and memory [42]. Experimentally, cholesterol depletion with methyl-β-cyclodextrine has decreased synaptic transmission and long-term potentiation in CA1 hippocampal regions in rats [49]. Impaired signaling and altered autophagy and apoptosis could be another effect of cholesterol depletion in neurons [50] by downregulating the AKT pathway and inactivating ERK phosphorylation in cultured hippocampal cells. 

The link between vascular disease and dementia is complex because aging, hypertension, atherosclerosis, or even amyloid deposition may lead to cerebrovascular dysfunction, alter the blood brain barrier, compromise the microenvironment in the brain parenchyma, and increase the vulnerability of certain regions such as the hippocampus, subcortical white matter, or neocortex to ischemic-hypoxic brain lesions [51,52]. In fact, vascular dementia is second in frequency only to Alzheimer’s disease [53] and incident stroke approximately doubles the risk of dementia in the elderly [54,55]. Indeed, observational studies have documented an association between elevated levels of serum total and LDL-cholesterol and amyloid deposition [56] and increased risk of stroke and dementia [57]. 

However, no randomized controlled trials have shown that statin therapy can prevent dementia in elderly individuals at risk of vascular disease [51,58]. In the Heart Protection Study, treatment with simvastatin for five years did not prevent cognitive decline in patients with cardiovascular disease [59]. Similarly, pravastatin treatment for 3 years in the PROSPER trial did not prevent cognitive decline in older adults with vascular risk factors [60]. 

These findings raise the question of how to treat elderly patients with stroke who have low LDL-C levels or in whom LDL-C levels are not significantly involved in the pathogenesis of stroke, such as cardioembolic stroke without significant carotid atheromatosis, without harming their cognitive functions as side-effect of the statin use.

Since the discovery of hydroxy-methylglutaryl-coenzyme A (HMG-CoA) reductase inhibitors (generically known as statins) starting with the work of Akira Endo, who identified mevastatin produced by Penicillium citrinum as a defense mechanism [61], HMG CoA reductase inhibitors have become a rapidly developing group of drugs increasingly prescribed to patients for prevention of vascular events. Over 32 million Americans are currently on statin therapy and approximately 56 million people (24% of the USA population) are eligible for statin treatment [21], a situation which led atorvastatin to be the second most prescribed drug in the USA [21]. Of the patients on statin therapy, 41% are 75 years and older [62]. Indeed, in our group of patients as well, more than one half were on statin therapy, mainly on atorvastatin.

A series of mild side effects, such as mild mental confusion and euphoria, were described already since phase I clinical trials of atorvastatin [63]. Post-marketing reports continued to describe transient and reversible short-term memory losses after statin therapy, mainly related to atorvastatin and simvastatin, although voluntary adverse event reports captures at most 10% of events [21]. By extrapolation, Sahebzamani and colleagues assumed that there is an incidence of between 3000 and 30,000 cases of cognitive impairment each year associated with these two statins in the USA [64]. A double-blind, placebo-controlled trial demonstrated detrimental effects of lovastatin on attention, working memory, and overall mental efficiency [65], while another double-blind, randomized trial found a significant impairment of memory and of the ability to learn from prior experiences in patients taking simvastatin as compared to placebo [66]. Subsequently, the 2013 American College of Cardiology/American Heart Association guidelines reviewed this safety issues and concluded that there is no consistent evidence for the adverse effect of statins on cognition [21]. However, their findings were based mainly on the Justification for the Use of Statins in Primary Prevention (JUPITER) trial, Pravastatin in Elderly Individuals at Risk of Vascular Disease (PROSPER) trial, and Heart Protection Study (HPS) trial, neither of which did list cognitive dysfunction as primary or secondary outcome [21]. As such, no detailed neuropsychological evaluation was performed, which is necessary to detect cognitive side effects. Even in our groups of patients, the difference in the MMSE score between the two groups failed to reach statistical significance while other, more complex scales, were able to detect subtle differences. In addition, PROSPER used pravastatin, which does not cross the blood brain barrier and the statin doses were mostly not as high as current guidelines recommend [67]. Possible poor metabolism in the elderly could lead to even higher exposure to this drug category [21]. Finally, most of the randomized controlled trials tend to exclude patients with a history of psychiatric diseases or alcohol abuse [68].

Several mechanisms have been proposed to explain the association between cognitive impairment and statins. HMG CoA reductase inhibitors can be subdivided into lipophilic and hydrophilic statins. Lipophilic statins, such as simvastatin or atorvastatin, can cross the blood brain barrier, while hydrophilic ones, such as pravastatin and rosuvastatin, do not [69]. Because the brain has the capacity of synthesizing its own cholesterol, mainly in astrocytes, it does not depend on cholesterol from the systemic circulation [70]. However, HMG-CoA reductase catalyzes the conversion of HMG-CoA to l-mevalonate and coenzyme A. As such, inhibition of HMG CoA reductase can prevent endogenous production of cholesterol. Statin treatment does not abruptly disrupt brain cholesterol homeostasis, but chronic statin treatment may be associated with significant reductions in CNS cholesterol [71] which results in neuronal degeneration and clinically in cognitive impairment progressing to dementia. Mood disturbances, and impulsive behavior, other neuropsychological side effects of statins, could result from a lower central serotoninergic release, as suggested by Ainiyet and colleagues [72], as well as from a reduced number of serotoninergic receptors which would decrease synaptic binding and re-uptake of serotonin [73]. However, a recent retrospective study failed to show an increase in the risk of depression in patients on lipophilic statins [74]. In addition, lipophilic statins have been shown to increase the cerebral levels of inflammatory cytokines, like IL-1beta and TNF-alpha, suggesting that an enhanced inflammatory mechanism may alter the function of the neurons [75]. In support of the direct influence of the lipophilic statins on the brain cholesterol come the findings of more frequent associations of cognitive impairments with the recent use of atorvastatin and simvastatin [76,77], which are lipophilic statins, as well as the findings of Rojas-Fernandez and colleagues, who showed that switching from a lipophilic to a hydrophilic statin might resolve the cognitive impairment [78]. The differences between the two classes of statins in cardiac outcomes are also not settled: while a meta-analysis showed similar effects of the two types of statins in coronary artery disease [79], the lipophylic atorvastatin in 40 mg daily dose was superior in increasing left ventricular ejection fraction and in reducing markers of fibrosis in patients with heart failure as compared to the hydrophilic rosuvastatin in daily doses of 20 mg over 6 months [80]. 

The findings of our study are supported by a recent meta-analysis of 28 trials, which concluded that there is still uncertainty regarding statin efficacy and safety in older people, with a trend toward less reduction in vascular events (stroke, coronary revascularization or vascular mortality) in patients aged over 75 years without established atherosclerotic cardiovascular disease [81].

Studies have also addressed concerns regarding the possible cognitive adverse effects of the newer agents which allow for LDL-cholesterol lowering, such as ezetimibe and evolocumab. To date, the Improved Reduction of Outcomes: Vytorin Efficacy International Trial (IMPROVE-IT) showed that ezetimibe taken for 7 years had a similar neurocognitive safety profile as placebo [82]. Evolocumab was assessed in EBBINGHAUS (Evaluating PCSK9 Binding Antibody Influence on Cognitive Health in High Cardiovascular Risk Subjects), a sub-study of the FOURIER (Further Cardiovascular Outcomes Research With PCSK9 Inhibitors in Subjects With Elevated Risk) trial [83]. During a mean follow-up period of 19 months no significant changes in executive function, memory, or psychomotor speed could be discerned [84]. In addition, the EWTOPIA 75 trial which evaluated the effect of ezetimibe in primary prevention of cardiovascular events in elderly Japanese patients showed it to be efficient despite the issues of the study related to its open-label nature, premature termination, and problematic follow-up of patients [85]. The effect of low-dose statin plus ezetimibe remains to be evaluated [81].

### Limitations

First, the study was carried out on a small sample size, in patients whose cognitive status before being hospitalized for stroke is unknown. In addition, records of prior strokes are missing. However, by choosing patients of similar age, we tried to minimize the bias in this regard. 

Second, due to the fact that stroke itself can impair cognition, tests done early after stroke onset can yield false results. We tried to minimize this drawback by excluding patients with aphasia or motor impairments which could interfere with the completion of tests, as well as patients with an established diagnosis of dementia, and to include in the control group patients with similar stroke severity. To exclude such bias, patients should have been tested before the vascular event, which is more difficult to carry out by primary care physicians.

Third, the tests used, particularly the MMSE and MoCA tests, are screening tools that do not allow for detailed testing of the various cognitive domains. Therefore, statistical analysis for subtests, together with the small sample size, may be biased by type 1 errors. However, since it is these tests that we use in everyday practice, our psychologists are more familiar with them.

Fourth, we did not perform long-term follow-up and repeated testing, although given the exclusion criteria it is unlikely that the cognitive performances of our patients (aged over 65) would have improved significantly.

## 5. Conclusions

Our study, despite the small sample size and the lack of information regarding the cognitive performances of the patients before initiating statin therapy, suggests caution in prescribing these drugs to elderly, frail ischemic stroke patients with modest levels of LDL-C. This would be especially advisable if the stroke pathogenesis is not directly related to LDL-C, such as cardioembolic or even lacunar strokes. Cognitive assessment might help choosing between lipophylic and hydrophilic statins. We do not deny the important role of published guidelines and the many beneficial effects of statins in primary and secondary prevention of vascular events. However, we do believe that future updates should take into account the potential for cognitive impairment of very low serum LDL-cholesterol levels in elderly patients. Like in the MATCH trial [86], where dual antiplatelet therapy (shown to have synergistic effect in coronary heart disease) did not significantly lower the rate of ischemic stroke recurrence but increase the risk of cerebral bleedings, it may be that pushing the LDL-cholesterol targets toward 40 mg/dL may have significant cognitive side effects despite a robust reduction of the risk of ischemic vascular events.

## Figures and Tables

**Table 1:** Gender, risk factors, and clinical characteristics of the two groups of patients.

LDL-C LEVEL	LDL-C ≤ 70 mg/dL	LDL-C > 70 mg/dL	*p*
Gender–*N* (%)	Male—16 (55.2%)	Male—17 (58.6%)	*p* = NS
	Female—13 (44.8%)	Female—12 (41.4%)	
Systolic blood pressure (mean ± SD)	150.62 ± 22 mm Hg	160.76 ± 22.9 mm Hg	*p* = NS
Diastolic blood pressure (mean ± SD)	76.14 ± 7.65 mm Hg	83.31 ± 10.4 mm Hg	*p* < 0.005
Diabetes mellitus—N (%)	10 (34.5%)	11 (37.9%)	*p* = NS
Body mass index (mean ± SD)	23.92 ± 5.53	26.57 ± 4.35	*p* < 0.05

LDL-C = LDL-cholesterol; N = number; (%)—percentage; NS = non-significant difference; SD = standard deviation; *p* = significance of the difference. Frequencies were compared with the chi square test, while continuous variables (blood pressure and body mass index) were compared with the two-tailed independent samples t-test.

**Table 2:** Incidence of ischemic stroke subtypes and stroke severity in the high and low LDL-cholesterol groups of patients.

Stroke Subtype	LDL-C ≤ 70 mg/dL	LDL-C > 70 mg/dL	*p*
Thrombotic—*N* (%) NIHSS score (mean ± SD)	2 (6.9%) 8.00 ± 2.57	8 (27.6%) 8.37 ± 1.99	*p* = 0.058 *p* = 0.884
Embolic—*N* (%) NIHSS score (mean ± SD)	8 (27.6%) 7.25 ± 3.37	3 (10.3%) 8.30 ± 3.00	*p* = 0.132 *p* = 0.739
Lacunar—*N* (%) NIHSS score (mean ± SD)	13 (44.8%) 5.92 ± 2.06	11 (37.9%) 5.72 ± 1.90	*p* = 0.683 *p* = 0.811
Undetermined etiology—*N* (%) NIHSS score	6 (20.7%) 7.50 ± 2.42	7 (24.1%) 6.71 ± 0.95	*p* = 0.782 *p* = 0.483
Overall NIHSS score (mean ± SD)	6.75 ± 2.57	6.93 ± 2.10	*p* = 0.781

LDL-C = LDL-cholesterol levels; SD = standard deviation; *p*—statistical significance revealed by the chi square test; NIHSS = National Institute of Health Stroke Scale; N—number; (%)—percentage.

**Table 3:** Lipid profile in the two groups of 29 patients.

LDL-C LEVEL	LDL-C ≤ 70 mg/dL	LDL-C > 70 mg/dL	*p*
LDL-C (mean ± SD, in mg/dL)	60.97 ± 8.37	133.24 ± 27.39	*p* < 0.001
HDL-C (mean ± SD, in mg/dL)	46.10 ± 8.95	42.90 ± 12.21	*p* = 0.260
Triglycerides (mean ± SD, in mg/dL)	133.28 ± 39.78	168.59 ± 53.65	*p* = 0.006
Total cholesterol (mean ± SD, in mg/dL)	133.72 ± 16.06	209.86 ± 33.62	*p* < 0.001

LDL-C = LDL_cholesterol levels; HDL-C = HDL cholesterol levels, SD = standard deviation; *p* = statistical significance of the difference, as revealed by the independent samples t-test.

**Table 4:** MoCA total and subscale scores in the two groups of patients (mean scores and standard deviation).

LDL-C LEVEL	LDL-C ≤ 70 mg/dL (mean ± SD)	LDL-C > 70 mg/dL (mean ± SD)	*p*
MoCA total score	22.45 ± 2.55	25.31 ±2.39	*p* < 0.001
MoCA visuo-spatial/executive score	3.10 ± 0.49	4.24 ± 0.57	*p* < 0.001
MoCA naming score	2.62 ± 0.56	2.76 ±0.43	*p* > 0.05
MoCA attention score	3.21 ± 0.62	4.24 ± 0.92	*p* < 0.001
MoCA language score	2.41 ± 0.50	2.62 ± 0.49	
MoCA abstraction score	1.38 ± 0.56	1.66 ± 0.68	*p* = 0.05
MoCA recall score	3.48 ± 0.83	3.93 ± 0.79	*p* = 0.041
MoCA orientation score	5.52 ± 0.68	5.28 ± 0.65	*p* > 0.05

MoCA—Montreal Cognitive Assessment, LDL-C = LDL-cholesterol levels, *p* = statistical significance of the difference, as revealed by the independent samples t-test.

**Table 5:** ACE-III total and subscale scores in the two groups of patients (mean scores and standard deviation).

LDL-C LEVEL	LDL-C ≤ 70 mg/dL	LDL-C > 70 mg/dL	*p*
Addenbrooke test attention score (mean ± SD)	13.41 ± 2.26	15.59 ± 1.86	*p* < 0.001
Addenbrooke test memory score (mean ±SD)	15.41 ± 4.88	19.21 ± 4.48	*p* = 0.003
Addenbrooke test fluency score (mean ± SD)	8.69 ± 1.87	10.41 ± 2.02	*p* = 0.001
Addenbrooke test language score (mean ±SD)	17.00 ± 4.83	20.14 ± 4.59	*p* = 0.014
Addenbrooke test visuo-spatial abilities score (mean ± SD)	9.72 ± 1.94	13.03 ± 2.47	*p* < 0.001
Addenbrooke scale total score (mean ± SD)	64.24 ± 13.21	78.38 ± 13.29	*p* < 0.001

**Table 6:** Partial correlation of the MMSE, MoCA total and ACE-III total scores with LDL-C levels, nutritional status, systolic and diastolic blood pressure.

Control Variables	Neuropsychological Test Results	Parameters of Statistical AnalysisTitle	BMI	Systolic BP	Diastolic BP	LDL-C Levels (mg/dL)
Age of patient and NIHSS score	MMSE score	Pearson coefficient	0.328	0.135	0.188	−0.297
		Significance (2-tailed)	0.013	0.323	0.166	0.026
		df	54	54	54	54
	MoCA Total score	Pearson coefficient	0.331	0.139	0.293	−0.549
		Significance (2-tailed)	0.013	0.306	0.029	0.000
		df	54	54	54	54
	ACE-III Total score	Pearson coefficient	0.397	0.140	0.268	−0.585
		Significance (2-tailed)	0.002	0.302	0.046	0.000
		df	54	54	54	54

MMSE = Mini Mental State Examination test; MoCA = Montreal Cognitive Assessment test; ACE-III = Addenbrooke’s Cognitive Examination—version III; LDL-C = LDL-cholesterol levels; Significance = significance level detected by the 2-tailed Independent Samples *t*-test; NIHSS = National Institute of Health Stroke Scale. BMI—body mass index. BP – blood pressure

**Table 7:** Linear regression of the total ACE-III scores as dependent variable and age, BMI, diastolic BP, LDL-C, and NIHSS scores as independent variables.

Model	R Square Change	Change Statistics			Significance of F Change
		F Change	df1	df2	
1	0.388 ^a^	35.501	1	56	0.000
2	0.094 ^b^	9.973	1	55	0.003
3	0.138 ^c^	19.566	1	54	0.000
4	0.003 ^d^	0.397	1	53	0.531
5	0.002 ^e^	0.232	1	52	0.632

^a^ Predictors: (Constant), age of patient; ^b^ Predictors: (Constant), age of patient, body mass index; ^c^ Predictors: (Constant), age of patient, body mass index, LDL-C levels; ^d^ Predictors: (Constant), age of patient, body mass index, LDL-C levels, NIHSS score; ^e^ Predictors: (Constant), age of patient, body mass index, LDL-C levels, NIHSS score, diastolic BP.

## Data Availability

Study data are available upon request from corresponding author.
